# Supplementary material for: Validation of a multi-ancestry polygenic risk score and age-specific risks of prostate cancer: A meta-analysis within diverse populations
Source: eLife. 2022 Jul 8;11:e78304. doi: 10.7554/eLife.78304 (PMC9322982; doi:10.7554/eLife.78304)
Supplement: Figure 1—source data 1. — Results in men of European ancestry were meta-analyzed across Million Veteran Program (MVP), UK Biobank, and Mass General Brigham (MGB) Biobank. Results in men of African ancestry were meta-analyzed across MVP, California and Uganda Prostate Cancer Study (CA UG), Maryland Prostate Cancer Case–Control Study (NCI-MD), Men of African Descent and Carcinoma of the Prostate (MADCaP) Network, and MGB Biobank. Results in Hispanic men were from MVP. The PRS association for men in the 99–100% category was not assessed in the MGB Biobank and therefore was not included in the meta-analysis. In each replication study, PRS categories were determined based on the distribution in controls. ORs and 95% CIs were estimated from logistic regression models adjusting for age and principal components of ancestry. [file elife-78304-fig1-data1.docx]

**Figure 1 – Source data 1**

**Association between the multi-ancestry PRS and prostate cancer risk replicated in men from European, African, and Hispanic populations.** Results in men of European ancestry were meta-analyzed across MVP, UK Biobank, and MGB Biobank. Results in men of African ancestry were meta-analyzed across MVP, CA UG, NCI-MD, MADCaP Network, and MGB Biobank. Results in Hispanic men were from MVP. The PRS association for men in the 99-100% category was not assessed in the MGB Biobank and therefore was not included in the meta-analysis. In each replication study, PRS categories were determined based on the distribution in controls. ORs and 95% CIs were estimated from logistic regression models adjusting for age and principal components of ancestry.

|  | European Ancestry 22,049 cases, 414,249 controls | | | African Ancestry 8,794 cases, 55,657 controls | | | Hispanic 1,082 cases, 20,601 controls | | |
| --- | --- | --- | --- | --- | --- | --- | --- | --- | --- |
| PRS Category | OR | (95% CI) | P value | OR | (95% CI) | P value | OR | (95% CI) | P value |
| [0-10%] | 0.32 | (0.29 - 0.35) | 5.67E-126 | 0.37 | (0.32 - 0.43) | 4.17E-41 | 0.28 | (0.19 - 0.43) | 4.70E-09 |
| (10-20%] | 0.48 | (0.44 - 0.51) | 5.74E-77 | 0.55 | (0.48 - 0.62) | 9.75E-22 | 0.46 | (0.33 - 0.66) | 1.31E-05 |
| (20-30%] | 0.64 | (0.59 - 0.68) | 9.81E-35 | 0.66 | (0.59 - 0.74) | 1.61E-12 | 0.63 | (0.47 - 0.86) | 3.59E-03 |
| (30-40%] | 0.79 | (0.75 - 0.85) | 2.47E-12 | 0.70 | (0.62 - 0.78) | 1.16E-10 | 0.90 | (0.69 - 1.18) | 4.49E-01 |
| (40-60%] | 1.00 (reference) | | | 1.00 (reference) | | | 1.00 (reference) | | |
| (60-70%] | 1.33 | (1.26 - 1.40) | 8.90E-24 | 1.23 | (1.11 - 1.35) | 3.01E-05 | 1.21 | (0.94 - 1.55) | 1.32E-01 |
| (70-80%] | 1.62 | (1.54 - 1.71) | 3.26E-72 | 1.45 | (1.32 - 1.59) | 6.00E-16 | 1.84 | (1.48 - 2.30) | 6.12E-08 |
| (80-90%] | 2.18 | (2.08 - 2.29) | 2.28E-216 | 1.79 | (1.64 - 1.95) | 7.51E-40 | 1.87 | (1.50 - 2.33) | 2.30E-08 |
| (90-100%] | 3.78 | (3.62 - 3.96) | < 5.00E-324 | 2.80 | (2.59 - 3.03) | 1.38E-144 | 3.22 | (2.64 - 3.92) | 2.82E-31 |
| (99-100%] | 7.32 | (6.76 - 7.92) | < 5.00E-324 | 4.98 | (4.27 - 5.79) | 5.02E-95 | 6.91 | (4.97 - 9.60) | 1.45E-30 |
